# *In-situ* preparation of plant samples in ESEM for energy dispersive x-ray microanalysis and repetitive observation in SEM and ESEM

**DOI:** 10.1038/s41598-019-38835-w

**Published:** 2019-02-19

**Authors:** Eva Tihlaříková, Vilém Neděla, Biljana Đorđević

**Affiliations:** 1 Institute of Scientific Instruments of the Czech Academy of Sciences, Brno, 612 00 Czech Republic; 2 Department of Plant Biology, Mendel University in Brno, Brno, 613 00 Czech Republic

## Abstract

The Extended Low Temperature Method (ELTM) for the *in-situ* preparation of plant samples in an environmental scanning electron microscope enables carrying out repetitive topographical and material analysis at a higher resolution in the vacuum conditions of a scanning electron microscope or in the low gas pressure conditions of an environmental scanning electron microscope. The method does not require any chemical intervention and is thus suitable for imaging delicate structures rarely observable with common treatment methods. The method enables both sample stabilization as close to their native state as possible, as well as the transfer of the same sample from a low vacuum to an atmospheric condition for sample storage or later study. It is impossible for wet samples in the environmental scanning electron microscope. Our studies illustrate the high applicability of the ELTM for different types of plant tissue, from imaging of plant waxes at higher resolution, the morphological study of highly susceptible early somatic embryos to the elemental microanalysis of root cells. The method established here provides a very fast, universal and inexpensive solution for plant sample treatment usable in a commercial environmental scanning electron microscope equipped with a cooling Peltier stage.

## Introduction

The scanning electron microscope (SEM) has become a routine technique for the morphological study of a wide range of samples with a resolution up to nanometres; however most plant samples require at least dehydration prior to observation. Therefore, many techniques and methods for the preparation of biological samples have been developed but none of them are universally applicable and artefact-free^1^. In general, samples can be studied after removing or changing the liquids from the samples using various techniques^2^, after application of special chemical treatment^3^, in their frozen hydrated state (CryoSEM, Low Temperature SEM)^4^, or in their fresh and fully hydrated state in the environmental scanning electron microscope (ESEM)^5,6^.

Especially in plant samples, processing a specimen with common preparation techniques can cause different types of artificial changes in the structure arising from chemical fixation, the removal of water and the extraction of soluble components during chemical fixation and drying via solvents. Moreover, structural features of plants can cause a problem with common preparation protocols. The external surface of most plant tissue is protected by a highly water- and chemical-resistant cuticle and the tough cell wall act as a barrier to reagents, and frequently dissection is needed to allow chemical treatment^7^. Cross-linking of the cell wall during the fixation process is less effective due to low protein content so the mechanical strength of the tissue is removed and samples are easily damaged in handling^1^. The classic preparation method based on using fixatives, dehydration with organic solvents and critical point drying is not suitable for wax observation that is dissolved^1^.

Currently, a very popular technique for electron microscopy of biological samples is a Low Temperature SEM (LTSEM) or a CryoSEM, which allows the preservation and recording of biological samples in a fully hydrated and chemically unmodified state. These techniques involve the study of samples at temperatures between −100 °C to −175 °C. The preparation of frozen-hydrated samples involves following the operational phases: cryofixation, freeze-drying of fracturing and, if necessary, also coating^8^. The LTSEM/CryoSEM is demanding in terms of the specific hardware composed of cryo-preparation equipment and an SEM specimen stage cooled with liquid nitrogen. Although most artefacts characteristic for a dry specimen were eliminated, the LTSEM/Cryo SEM has its own specific artefacts arising from the behaviour of the water during cryofixation, freeze drying and specimen transfer^9^. A comparison of the ability of a low temperature method for ESEM (LTM), the CryoSEM and optical microscopy to image the early somatic embryo surface microstructure covered with a very fine extracellular matrix has been published^10^.

The direct study of fully hydrated or electrically non-conductive dry biological samples, without the necessity of covering their surface with a conductive layer is also possible in a high-pressure environment in the ESEM^11,12^. Observation of fully hydrated biological samples in the ESEM can be limited with a lower resolution in comparison with the SEM and low feasibility for additional analysis or repetitive imaging due to their collapse during or after observation. Moreover, susceptible biological samples which need to be fully hydrated tend to be easily damaged due to the influence of free radicals, local heating and drying^13^.

In order to eliminate problems associated with the observation of wet samples, The LTM for ESEM has been developed^13^. This method has been used in the study of plant samples in many studies^14–16^. Recently, it has also been successfully applied in the study of small animals^17^. The LTM for ESEM is based on the low temperature stabilization of a sample using a mutual combination of optimized speeds of gas pumping and sample cooling up to a temperature of −20 °C and pressure of 200 Pa of water vapour instead of the observation of hydrated samples in ESEM (temperature usually from 0 °C to 5 °C and water vapour pressure from 613 Pa to 866 Pa). The low temperature stabilisation of samples benefits from the assumption that the liquid solution inside the sample is unaffected due to its differential pressure contrary to the liquid water on the sample surface which is gently evaporated/sublimated. Moreover, plants are protected owing to their capability of producing components inhibiting ice formation or its growing such as polysaccharides in the cell wall^18^ and antifreeze proteins^19^. Although in some species the growth of ice crystals can occur, the cell walls resist collapse in the cellular volume, creating a divergence from the equilibrium^20^ and in combination with the high relative humidity in the ESEM, reduce the extent of dehydration.

An advantage of the LTM for ESEM is the capability of preserving sample surface morphology, increasing sample resistance to beam damage and the possibility of higher resolution observation. The LTM for ESEM works with temperatures that are reachable using standard equipment of the ESEM as a cooling Peltier stage in contrast to other low temperature methods (LTSEM/CryoSEM) which involve the use of additional expensive instruments^21^. Moreover, samples do not require any liquid substitution^22^.

Recent studies^23–25^ have shown that the ESEM and SEM can complement each other. While the ESEM can provide an image of the biological samples in their native state, the SEM can offer a high-resolution image of the treated samples. However, there is a gap between these techniques due to the impossibility of direct transfer between different environmental conditions and different sample preparation requirements. For this case, the LTM for ESEM has been newly extended into the ELTM for further benefits lying in the advanced preparation of plant samples for additional or later analysis in different SEM or ESEM microscopes. This paper proves the applicability of this method for observation of the identical sample in their hydrated state in ESEM and completely dried in SEM with a well-preserved surface morphology. It was not published up to date.

## Results and Discussion

Commercial ESEMs allow the observation of samples in different modes: (1) in their native-fully hydrated state (2) dried in low vacuum conditions without the necessity of conductive coating (3) dried and coated in high vacuum SEM conditions or without coating under low beam energies. A combination of sample observation in individual ESEM modes enables extending the range of obtainable information for correct evaluation of a sample microstructure or recognizing possible artefacts.

The application possibilities of the ELTM method presented in this paper to prepare plant samples for observation in various ESEM modes are presented in the following paragraphs.

### Fully hydrated samples in ESEM and after application of the LTM for ESEM

**Figure 1:**
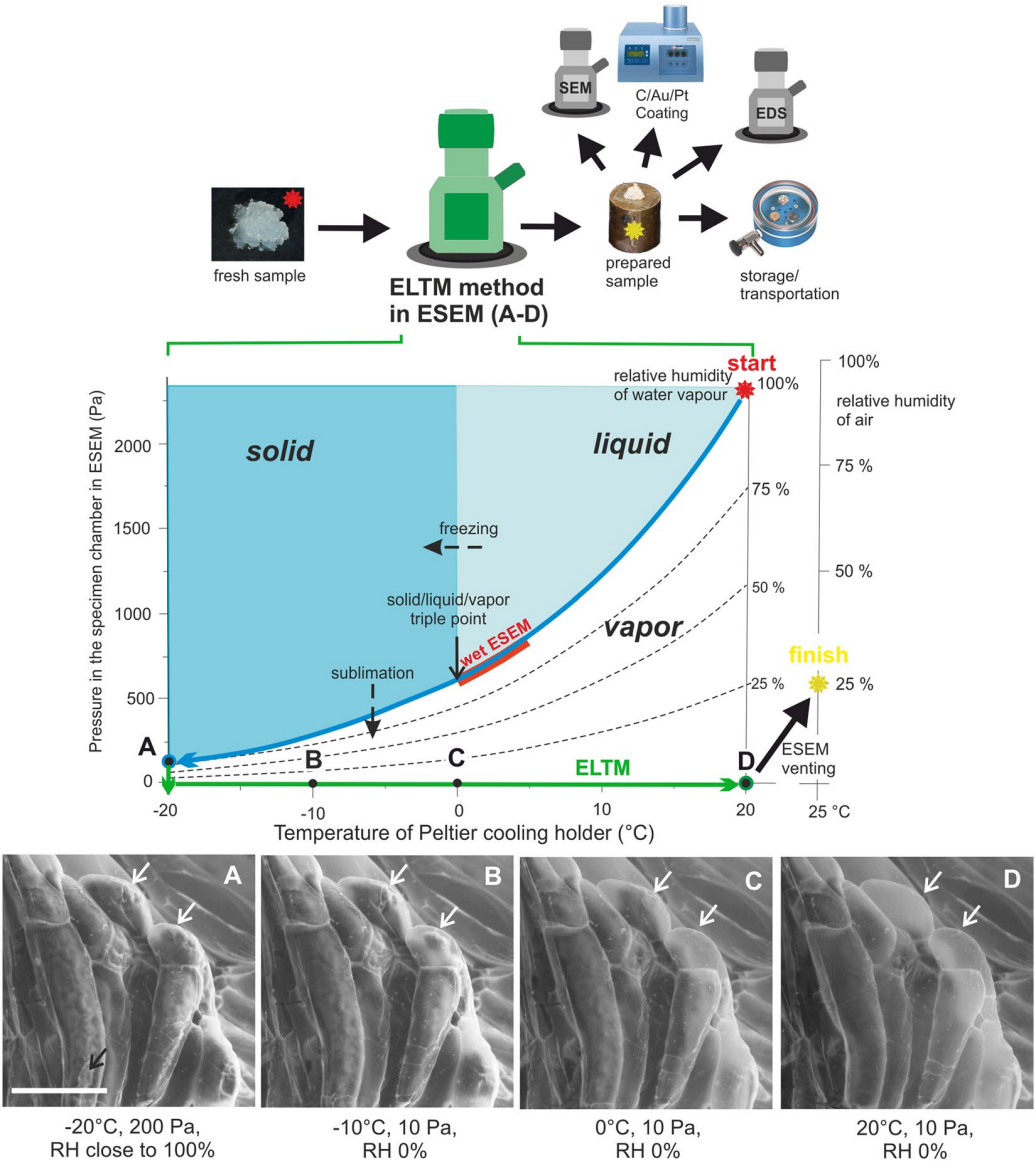
The upper schematic figure shows the introducing of the ELTM in an informative manner. The ELTM is realized during two steps inside the ESEM (green microscope). The steps corresponds with the two lines in the phase diagram (the 1^st^ step of the ELTM - freeze stabilization, blue line; the 2^nd^ step of the ELTM - water sublimation, green line). The state of the sample during the process is visible in images (**A**–**D**) placed below the phase diagram. The temperature and pressure conditions of images (**A**–**D**) correspond with the letters in the phase diagram. The bar is 50 μm. The white arrows point out places where the changes of the inner structures are well visible during the ELTM, the black arrow points out water residuum on the sample surface during observation in conditions close to 100% relative humidity.

The first step of the ELTM is mutual with the LTM and is based on specimen cooling up to −20 °C during the initial pumping of the microscope up to 200 Pa in the specimen chamber (Fig. 1 – phase diagram, blue arrow). Cooling and pumping run simultaneously. The rate of temperature and pressure changes are crucial parameters and must be set according to the type of sample. More susceptible samples with a higher content of water and with a thicker water layer on its surface need a decreasing of the cooling rate with a later start of pumping (around 0 °C), unlike robust samples with a low content of water and thick cuticles. However, for both cases, it should be ensured that most of water from the sample surface will be evaporated/sublimed and the maximum amount of water in the sample remains. Despite these dynamic changes, the sample is still in conditions close to 100% relative humidity in the specimen chamber.

The effect of the preparation procedure on sample morphology was evaluated by the imaging of an *Oxalis acetosella* leaf microstructure. The leaves are specific due to the low thickness associated with high sensitivity to drying out.

**Figure 2:**
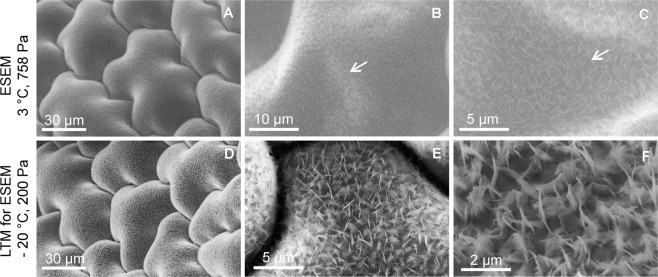
Wax microstructure on the surface of an *Oxalis acetosella* leaf observed in its fully hydrated state (**A**–**C**) and after application of the first step of the ELTM (**D**–**F**). The damage (indicated by white arrows) of the fully hydrated sample is evident in higher magnification (**B**,**C**) in contrast with the sample prepared using the LTM (**E**). The method also allows imaging of the high-resolution detail of wax structures (**F**).

At first, the sample was observed in its fully hydrated state which enables imaging the surface microstructure as close to its natural state as possible (Fig. 2A–C). However, the high-pressure conditions required in the specimen chamber of the ESEM cause electron beam diffusion in the gas, hence the signal to noise ratio in the detected signal decrease. In our study, this was compensated by using higher beam energy (20 keV). At higher magnification, the sample tends to collapse due to radiation damage and the impact of free radicals, see Fig. 2B,C - indicated by white arrows.

The surface morphology of the sample after the application of the first step of the ELTM evinces minimum changes (Fig. 2D–F). Moreover, the sample structure is freeze-stabilized with increased tolerance to radiation damage and allows a higher resolution to be reached. In addition to the study of a wax structure (Fig. 2F) which is prone to be melted by high energy beam electrons, an observation is possible instead Fig. 2C.

Now, the sample is well stabilized in conditions of low pressure and temperature, but not prepared for exposure to atmospheric pressure and ambient temperature. The second step of the ELTM must follow after observation which avoids sample collapse due to the changing of the thermodynamic conditions during the venting process of the ESEM.

### Extended LTM for ESEM

The second step of the ELTM lies in the gentle sublimation of the residual water from the sample inner structure and the transfer of the sample from the low pressure and temperature conditions to atmospheric conditions (Fig. 1 green arrow in the phase diagram). This process starts with a slow decreasing of pressure in the specimen chamber. When the smallest pressure, reachable in ESEM mode (approx. 10 Pa), is achieved the sample temperature can be slowly increased from −20 °C up to 20 °C. An increase in the sample temperature at low gas pressure allows decreasing the relative humidity (RH), thorough removing of the water from the sample and preventing water condensation on the sample surface after opening the specimen chamber of the ESEM. The sample processing with the ELTM is fully realised *in-situ*, inside the specimen chamber of the ESEM, as is visible in the schematic figure in Fig. 1 (above the diagram). The phase diagram of water in Fig. 1 shows temperature/pressure areas where the ELTM is realized from the start of the sample processing to its finish. Changes of the sample were monitored during the ELTM process and are visible on the sample of the suspensor cells of *Abies alba*, see Fig. 1A–D. The letters also correspond to letters in the phase diagrams. Figure 1A depicts the sample in the first step of the ELTM. The hydrated state of suspensor cells (white arrows) as well as the small rest of the ice on the sample surface (black arrow) is well visible there. During the second part of the ELTM (green arrow in the phase diagram) the sublimation is in the ascendant and the inner structure became less visible. If the sample temperature increases sufficiently slowly, minimal changes to the sample morphology are evident, see Fig. 1B–D. Images of similar samples in the wet state in ESEM and light microscopy were presented earlier^13^.

Finally, the sample can be moved out from the microscope and stored or used for any further analysis. The prepared samples are usually very fragile and the manipulation can cause artefact or damage formation. The sample can be placed on a thin glass coverslip before mounting on the cooled Peltier sample holder. It enables simple manipulation with the prepared sample and prevents artefact formation.

### Repetitive observation of biological samples in the ESEM

**Figure 3:**
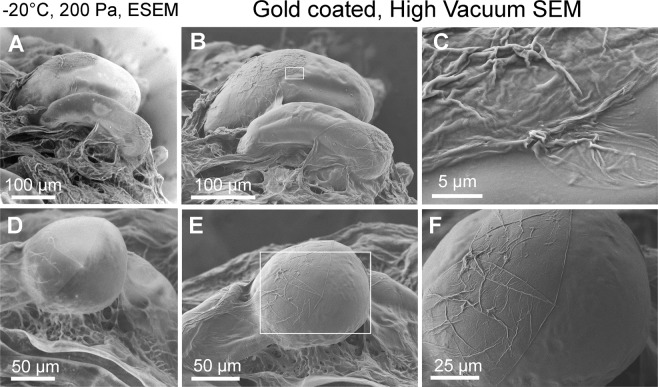
Samples of early somatic embryos of *Picea abies* observed during different steps of the ELTM. (**A**,**D**) Non-commercial ESEM AQUASEM II and conditions of 100% relative humidity. (**B**–**F**) SEM JEOL 6700 F with a high vacuum in the specimen chamber. The white arrows indicate the extracellular matrix.

Samples prepared using the ELTM are dry, with their surface morphology preserved in a state approximate to a fresh state and ready for handling for the purpose of further observation, analysis etc. The possibility to repetitive observation of the identical sample after its observation in the ESEM was demonstrated on early somatic embryos of *Picea abies* in Fig. 3. At first, samples were observed in a freeze stabilized state in conditions of −20 °C and 200 Pa of water vapour. The results show an absence of extracellular ice in all tissues, however achieving a high-resolution image is still limited, see Fig. 3A,D. After finishing the ELTM, samples were removed from the microscope, mounted on double-sided carbon tape, sputter coated with a thin layer of gold and observed in the SEM (10^−5^ Pa). The following SEM observation provides a more detailed view of the surface microstructure with higher resolution, see Fig. 3B,C,E,F.

The results in Fig. 3 provide tangible evidence that the application of ELTM enables observing the identical sample in both a hydrated and dry state with well-preserved surface micromorphology. The benefit of this method is also the ability to preserve delicate structures such as extracellular matrix (ECM) without any chemical treatments. The ECM is a thin membranous layer on the plant cell surface, see Fig. 3 indicated by arrows. Different preparation techniques (lyophilisation, glycerol substitution, liquid nitrogen substitution, chemical fixation and critical point drying) have been tested in the past but techniques that can avoid artefact formation were not found^26^. Preparation using the ELTM enables observing the ECM without common artefacts such as creating fibrillary structures as well as damage and hole creation. Structural arrangement of the ECM on the cell surface may play significant roles in morphogenical processes. Morphogenical observation of the samples prepared using the ELTM brings new results and possibilities to its accurate description. Moreover, the high resistance of the sample to repeated pressure changes in a range from atmospheric pressure to the vacuum obtainable during coating and SEM observation was noted after preparation using the ELTM.

### Energy dispersive x-ray microanalysis in ESEM

An application of the ELTM as a preparation method of plant samples for elemental microanalysis was demonstrated on a sample of cannabis root grown in a nutritional medium supplemented with Pb^2+^. In this case, after application of the ELTM, samples were positioned on a carbon stub with regards to the requirements of energy dispersive x-ray microanalysis and analysed in low vacuum conditions to prevent sample charging. Material contrast was observed using the self-designed YAG backscatter electron detector^27^ and analysis was realised using an x-ray cone to minimize beam electron scattering in the gas.

**Figure 4:**
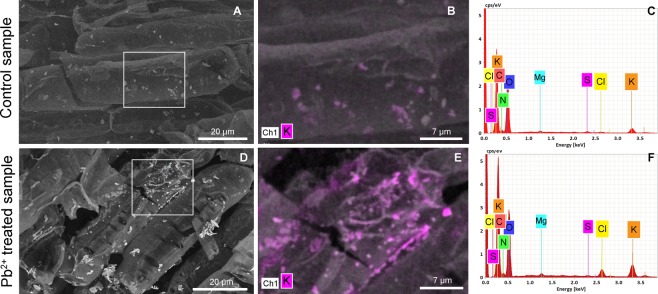
Cells of cannabis roots cultivated in a heavy metal-free medium (**A**–**C**) and exposed to Pb^2+^ (**D**–**F**). The material contrast imaged in backscattered electron images (**A**,**D**) shows specific element accumulation. Fragments with a map localization of K accumulation (**B**,**E**) and corresponding EDS spectra (**C**,**F**).

A significant impact of Pb^2+^ treatment was clearly visible via the material contrast of the root cells (Fig. 4D) where a specific element was accumulated between the cell borders in comparison with the control sample (Fig. 4A). A semi-quantitative analysis of several areas of root sections with qualitative x-ray mapping was combined. To describe the impact of Pb^2+^ treatment on the cannabis root, element localisation and chemical composition of samples were studied. Although no accumulation of Pb^2+^ was found, a significant variation in the concentration of K and Cl was observed in Pb^2+^ treated samples (Fig. 4E,F) in comparison with the control (Fig. 4B,C). The analysis confirmed that the elemental distribution of the described component was altered by metal treatment and was due to the affecting of the plant mineral metabolism and induced changes in the nutrient balance.

The sample prepared using the ELTM was observed in conditions of ambient temperature and a low-pressure environment in the specimen chamber of the ESEM. Positive ions generated by electron-gas interactions allow the elimination of charging the electrically non-conductive sample without the necessity of their conductive coating. Thus, the samples prepared using the ELTM in combination with the low-pressure environment are highly suitable for energy dispersive x-ray microanalysis. Generally, the use of the energy dispersive x-ray spectrometer (EDS) in ESEM is possible, but the results are strongly influenced by the scattering of primary electrons with gas. The high-pressure environment can cause the degradation of the effective spatial resolution, absorption of low energy peaks and the spectrum can be extended with X-rays from the gas, so the use of as low a pressure as possible is recommended. Owing to the ELTM, the EDS analysis could be realised in conditions of 150 Pa of water vapour in which the contribution of a significant artefact is strongly decreased.

## Conclusions

Our paper underlines the wide usability and advantages of the ESEM which can be used not only as a tool for the imaging of conventionally treated or highly susceptible fresh biological samples^28,29^ and polymers^30^, but also for fast, effective and inexpensive *in-situ* preparation allowing repetitive observation and elemental analysis of plant samples using our new ELTM. The *in-situ* preparation process can be controlled and directly modified according to sample specificity. It allows the amount of sample handling to be reduced to the indispensable minimum, hence contamination, damage or artefact formation is minimalized. Owing to the absence of chemical treatment, the ELTM is highly suitable for EDS microanalysis^31^ or observation of specific traits that can be damaged during conventional preparation methods. Sputter-coated free samples analysed under increased gas pressure conditions (up to 300 Pa) in the ESEM may also allow the presence of lower concentrated elements to be revealed, whose signal would be absorbed by the conductive layer. The quality of images is strongly dependent on the working conditions; however, a high variability of ESEM parameters, such as temperature, freezing velocity and humidity, can be found and set. Although this method has many limitations, the surface microstructure is well preserved with minimum artefacts and without expensive and time-consuming chemical treatments. The ELTM method can be applied in any commercial ESEM equipped with a cooled Peltier stage and routinely used for the imaging of plant samples in a higher resolution.

## Methods

### Plant material and culture conditions

The embryogenic tissue of silver fir (*Abies alba Mill*.) was initiated from immature zygotic embryos of cones from open-pollinated trees. Immature seeds were surface-sterilized for 10 min in 10% (v/v) H_2_O_2_ and then rinsed several times with sterile distilled water. The immature cones of *Abies alba* were collected on 26 July 2006 in the Dobroč primeval forest. The cultures were maintained in darkness at 25 ± 1 °C and at 2-3 week intervals transferred to a fresh medium.

The embryogenic culture of Norway spruce (*Picea abies (L*.*) Karst*.) collected by Plant Biology, Mendel University (Brno) was originally isolated from a mature zygotic embryo of a tree of spruce mountain climate type No. 12345 from an experimental area located in the Beskydy Mountains, Moravia, Czech Republic. The culture of embryonic tissues was maintained on medium designated LP/2 in90 mm diameter Petri dishes with 9 µM 2,4-D and 4.4 µM BAP. The embryogenic tissues from the upper parts of their aggregates (2.5–5.0 mg) were sub-cultured in 10–14 days periods. The cultures were incubated in the dark at 23 ± 1 °C.

For obtaining hemp plantlets for *in vitro* multiplication, seeds of the Bialobrzeskie variety obtained from Agritec Plant Research Institute Ltd., Šumperk, Czech Republic were used. The seeds were surface sterilized with 6% sodium hypochlorite for 8 minutes and afterwards rinsed three times with sterile distilled water. The sterilized seeds were germinated on half-strength MS medium supplemented with 29.2 mM sucrose and 0.65% agar. The pH value of the media was adjusted to 5.8 before autoclaving at 121 °C, 100 kPa, for 20 min. The seeds were maintained in a cultivation room under 18/6 light dark cycle at 24 ± 2 °C. The shoot tips (approx. 2 cm in length) were taken from 10-14-day-old plantlets and placed on a cultivation medium enriched with full-strength MS salts, 87.6 mM sucrose, 0.8% agar and 2 µM meta-topolin. The pH value of the media was adjusted to 5.7 before autoclaving. The standard cultivation medium was used as a control and experimental variants were supplemented with 750 µl Pb-EDTA. Explants were cultivated for one month on nutritional medium under 18/6 light dark cycle at 24 ± 2 °C when the effect of Pb^2+^ on the root growth was evaluated. The roots were sectioned at a distance of 1.2–1.5 cm from the apex where the root cells are already developed and this part mostly contributes to the uptake elements^32^.

### Environmental scanning electron microscopy

Environmental scanning electron microscopy micrographs were obtained with a non-commercial ESEM AQUASEM II and ESEM Quanta 650 FEG equipped with a Bruker QUANTAX EDS XFlash 6 detector. Fresh plant samples were sectioned approximately to 4–8 mm^2^, placed into a drop of water on the Peltier cooling stage equipped with a special flat cylindrical brass sample holder. In the case of observation of samples in their fully hydrated state, the sample temperature was 3 °C and 760 Pa of water vapour, the accelerating voltage 20 kV, the probe current 80 pA and the environmental distance between the sample surface and the second pressure limiting aperture was 8.5 mm.

In the case of the LTM for ESEM, the accelerating voltage was 10 kV, the probe current was 50 pA and the sample distance between the sample surface and the second pressure limiting aperture was 8.5 mm.

### Elemental analysis

The working conditions were: the accelerating voltage 10 kV, the probe current 100 pA, pressure of water vapour 150 Pa, working distance 10 mm. Parameters used for X-ray mapping were: image resolution 200 × 200 pixels, dwell time per pixel 5 ms, magnification 1000x.

### Scanning electron microscopy

Observation of dry samples was realized using an SEM JEOL 6700F and conventional vacuum 10^−5^ Pa. Samples were placed on a carbon tape and coated with gold using a Sputter Coater Q150 (Quorum Technologies).
